# Super-resolution microscopy reveals a preformed NEMO lattice structure that is collapsed in incontinentia pigmenti

**DOI:** 10.1038/ncomms12629

**Published:** 2016-09-02

**Authors:** Janine Scholefield, Ricardo Henriques, Anca F. Savulescu, Elisabeth Fontan, Alix Boucharlat, Emmanuel Laplantine, Asma Smahi, Alain Israël, Fabrice Agou, Musa M. Mhlanga

**Affiliations:** 1 Faculty of Health Sciences, Division of Chemical Systems and Synthetic Biology, Institute of Infectious Disease and Molecular Medicine, University of Cape Town, Anzio Road, Observatory, Cape Town, Western Cape 7925, South Africa; 2 Gene Expression and Biophysics Group, CSIR Synthetic Biology ERA, Pretoria 0001, South Africa; 3 Quantitative Imaging and Nanobiophysics Group, MRC Laboratory for Molecular Cell Biology and Department of Cell and Developmental Biology, University College London, Gower Street, London WC1E 6BT, UK; 4 Chemogenomic and Biological Screening Core Facility, Institut Pasteur, Center for Innovation and Technological Research (Citech), Departments of Cell Biology and Infection and of Structural Biology and Chemistry, 25/28 rue du Dr Roux, 75724 Paris cedex 15, France; 5 Laboratory of Signaling and Pathogenesis, CNRS, UMR 3691, Institut Pasteur, 75724 Paris cedex 15, France; 6 INSERM U1163-Université Paris Descartes-Sorbonne Paris Cité, Institut Imagine, Hôpital Necker-Enfants Malades, 24 Boulevard du Montparnasse, Paris 75015, France; 7 Faculdade de Medicina, Gene Expression and Biophysics Unit, Instituto de Medicina Molecular, Universidade de Lisboa, Lisbon 1649-028, Portugal

## Abstract

The NF-κB pathway has critical roles in cancer, immunity and inflammatory responses. Understanding the mechanism(s) by which mutations in genes involved in the pathway cause disease has provided valuable insight into its regulation, yet many aspects remain unexplained. Several lines of evidence have led to the hypothesis that the regulatory/sensor protein NEMO acts as a biological binary switch. This hypothesis depends on the formation of a higher-order structure, which has yet to be identified using traditional molecular techniques. Here we use super-resolution microscopy to reveal the existence of higher-order NEMO lattice structures dependent on the presence of polyubiquitin chains before NF-κB activation. Such structures may permit proximity-based *trans*-autophosphorylation, leading to cooperative activation of the signalling cascade. We further show that NF-κB activation results in modification of these structures. Finally, we demonstrate that these structures are abrogated in cells derived from incontinentia pigmenti patients.

## Abstract (web summary)

NEMO is a member of the IKK complex that binds ubiquitin, involved in NF-κB signalling and proposed to form higher order structures. Here the authors use super-resolution microscopy to detect the presence of NEMO lattices in cells, that are modified by NF-κB treatment and abrogated by mutations affecting NEMO ubiquitin binding.

## Graphical abstract

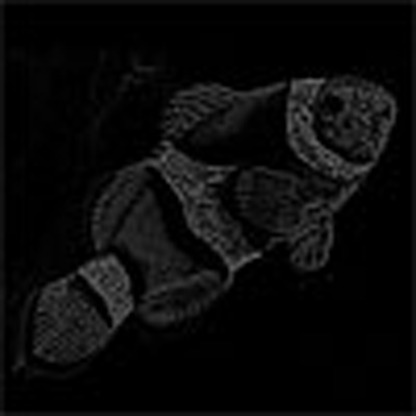

The nuclear factor-κB (NF-κB) signal transduction pathway plays important roles in mammalian immune and inflammatory responses to injury and infection, as well as cell proliferation, differentiation and survival^1^. Indeed, several genetic diseases result from the functional alteration of key components of NF-κB signalling^2^. Among these are the various rare X-linked human diseases, including incontinentia pigmenti (IP, OMIM # 308300) and immunodeficiency with or without anhydrotic ectodermal dysplasia (ID OMIM# 300291).

On exposure to various stresses or external stimuli, cell surface receptors activate the inhibitor of κB kinase (IKK) complex, permitting nuclear entry of NF-κB and transcription of hundreds of target genes^3^. This classical view of NF-κB signalling has invoked the notion of sequential activation via posttranslational and conformational modifications to individual proteins. However, a shift in our understanding of the critical fine-tuning of this pathway is emerging from increasing evidence of ‘open-ended' higher-order structures^4^. The close proximity of regulatory proteins in these structures would enable spatio-temporal control of signal amplification enhancing efficiency of subsequent catalytic reactions. Such cooperativity would be greatly facilitated if a lattice-like structure pre-existed within the cell, before signal induction^5^.

Higher-order structures have been shown for the tumour necrosis factor receptor-associated factor 6, responsible for the Toll-like receptor/interleukin (IL)-1-dependent signal transduction of NF-κB (ref. 6). By elucidating the crystal structure of tumour necrosis factor receptor-associated factor 6, Wu and colleagues revealed the presence of a scaffolding network of oligomerized proteins at the plasma membrane. The authors proposed that such higher-order oligomerization facilitates the regulation of signal transduction by enhanced proximity-based activation and autoubiquitination.

A similar model has been hypothesized for the IKK complex^7^, which represents a critical checkpoint in the canonical activation of NF-κB. A key factor in the IKK complex is the NF-κB essential modulator protein (NEMO, also known as IKKγ), responsible for the regulation of the catalytic IKK subunits^8^,^9^. Indeed, hints of a higher-order oligomeric structure emerged in a previous study labelling NEMO following IL-1 stimulation^10^. However, confocal microscopy revealed no evidence of a preformed higher-order structure. Structurally, NEMO consists of an elongated and flexible coiled-coil protein, which can form a stable dimer in solution and putatively polyubiquitin-mediated higher-order oligomers *in vitro* (trimer, tetramer and multimers) and *in vivo* via its coiled-coil domains^11^,^12^,^13^. Furthermore, the catalytic IKKβ subunits from two different species were recently crystallized^14^,^15^ and both were shown to form a stable dimer with a trimodular organization. Strikingly, crystallization studies demonstrated that different oligomers of IKK kinases can be formed in the asymmetric unit of the crystal lattice through specific contacts between kinase domains, the predominant assembly forming octameric (tetramer of IKK dimers), hexameric (trimer of IKK dimers) and dimeric structures. The existence of these higher-order oligomers, which are largely undetectable in solution, has been proposed to provide a possible mechanism of cooperativity for IKK activation via IKKβ catalysed autophosphorylation^7^. Their existence would implicate the higher-order structure directly in NEMO function. Furthermore, the loss of this higher-order structure could be a key mechanism by which NEMO signalling could be compromised.

We and others have shown that hypomorphic mutations in NEMO that lead to impairment, but not abolition of NF-κB signalling, are associated with the less clinically severe anhydrotic ectodermal dysplasia syndrome, whereas amorphic NEMO mutations that completely abolish NF-κB activation are associated with IP in females and *in utero* lethality in males^16^. Most IP patients (70–80%) bear a complex genomic rearrangement, leading to the deletion of exons 4–10 (ref. 17). This recurrent NEMO rearrangement results in the synthesis of a truncated non-functional protein, which is devoid of its two carboxy-terminal ubiquitin binding domains (NOA/ubiquitin-binding domains in ABIN and NEMO (UBAN) and zinc finger (ZF)), but still able to interact with the IKKs. Other IP-associated mutations, in contrast, appear more subtle, such as the A323P missense mutation. Yet, this single base-pair change confers a severe defect to the NEMO protein, affecting both its ability to oligomerize and bind polyubiquitin^18^. An open question is whether these IP causing mutations compromise the putative higher-order structure of NEMO and thus interfere with the essential mechanism required for functional NEMO signalling.

With several lines of evidence pointing towards a functionally relevant higher-order structure of NEMO, it remains to be identified *in cellulo*, due in part to the limited ability of experimental techniques at our disposal.

The existence of a higher-order structure would be best established by directly visualizing its nanoarchitecture in cells. However, cryo-electron microscopy techniques involve complex sample manipulations that may result in the collapse of these fragile structures making the approach unsuitable for visualization. In light microscopes, the diffraction limit of light restricts the spatial resolution preventing the accurate observation of molecular assemblies below 200 nm. However, recent advances in super-resolution methods have allowed the classical limit to be breached and resolutions of 10–20 nm to be routinely achieved via fluorescent light microscopy^19^.

Here we use super-resolution microscopy to reveal the putative higher-order structure of NEMO in non-stimulated cells, both in live-cell and fixed conditions. We demonstrate that abrogation of non-covalent polyubiquitin chains or IKK:NEMO binding leads to the disruption of these higher-order structures. Surprisingly, the largest structures represent a very small fraction of total NEMO higher-order structures in cells, yet even a modest removal of this subset results in defective NEMO signalling. Furthermore, we provide quantitative evidence for the modification of these structures on NF-κB stimulation. Finally, we show that IP patient-derived cells lack such higher-order lattices, providing a molecular understanding of their pathology. Collectively, our data demonstrate the importance of higher-order oligomerization of NEMO in the NF-κB signalling response and emphasize how super-resolution microscopy can provide novel insights into the single-molecule mechanisms of genetic disorders.

## Results

### Super-resolution reveals a higher-order structure of NEMO

**Figure 1:**
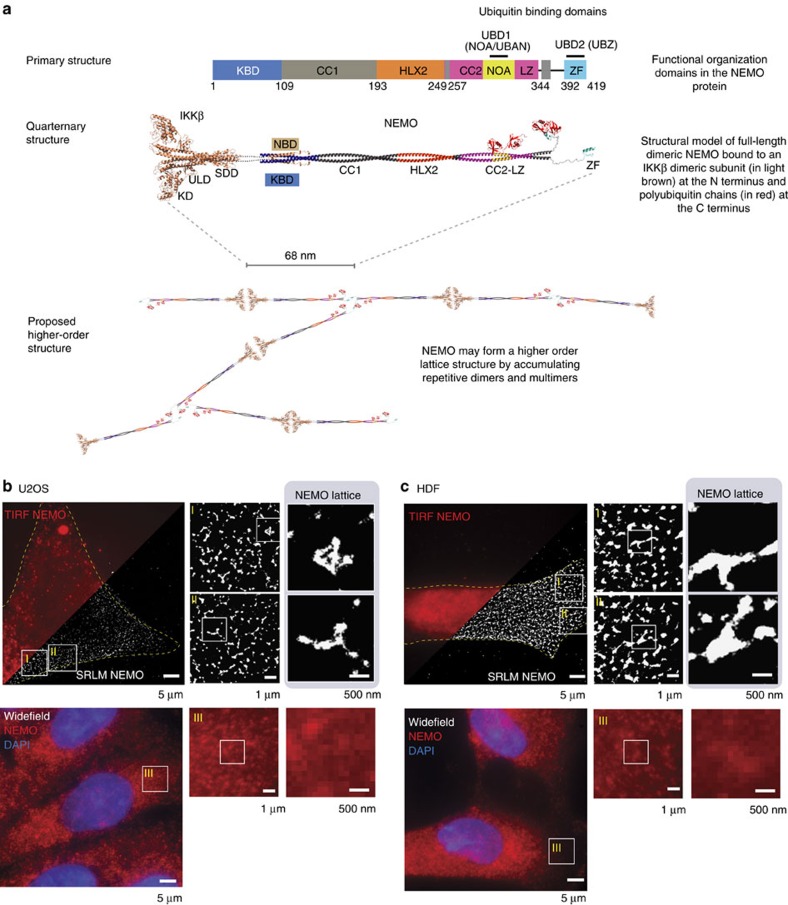
Depiction of higher-order NEMO lattice structure. (**a**) Functional domains and model of the full dimeric IKKβ-NEMO complex showing the highly elongated shape of the potential higher-order structure. All crystal and NMR structures of the NEMO domains are depicted in bright colours, whereas those modelled are in grey. The two ubiquitin-binding domains (NOA and ZF) are shown as complexes with K63 di-Ub chains (red). The dimeric structure of IKKβ (light orange) consisting of its three sub-domains (KD, ULD and SDD) interacts via its C-terminal extremity (NBD, light orange) with the N-terminal kinase binding domain (KBD, blue) of NEMO. The dotted lines denote the short IKKβ fragment (residues 666–704) and the human NEMO (1–48), which are lacking in the crystal structures as described in Methods. The structural model of full-length NEMO bound to IKK and polyubiquitin chains is used to propose a higher-order lattice structure by accumulating repetitive base units. SRLM of NEMO in U2OS cells (**b**) and primary fibroblasts (**c**) reveals extensive branching of NEMO lattice. Top left micrograph is an overlay of TIRF and SRLM images showing a yellow dotted outline of the cell boundary. Box I and II are magnified from the left image and then shown with further boxed magnification. (All subsequent SRLM images follow the same format, unless otherwise indicated.) Bottom left micrograph represents cells from the same coverslip imaged using conventional fluorescence microscopy. Box III is representative of the equivalent widefield magnification shown in Box I and II above, as is the subsequent magnified inset. HLX2, helical domain 2; KD: kinase domain; NBD, NEMO binding domain; NOA: NEMO, OPTINEURIN and ABIN domain; SDD, α-helical scaffold/dimerization domain; ULD, ubiquitin-like domain; ZF, zinc finger.

Biochemical and structural data reveal that NEMO forms an elongated dimer^20^, which can act as a regulatory scaffold to activate the catalytic IKK subunits in a polyubiquitin binding-dependent manner (Fig. 1a). X-ray crystallography data^14^ from IKKβ had further suggested that this higher-order NEMO-IKK platform would form a lattice organization in the order of 500 nm or greater, composed of ∼68 nm base units. We reasoned that given the resistance of the proposed higher-order structure to biochemical isolation, it would be best visualized directly using super-resolution localization microscopy (SRLM) techniques such as photoactivated localization microscopy (PALM)^21^ and stochastic optical reconstruction microscopy (STORM)^22^-based methods. SRLM has been used to reveal novel structural information at the nanoscale level for a variety of biological applications (reviewed in ref. 23), including disease pathogenesis^24^. Therefore, we believed this to be the best approach to investigate whether the NEMO-IKK unit is assembled into higher-order structures. For high-precision fixed cell super resolution, we opted for direct STORM (dSTORM)^25^ allowing us to survey endogenous NEMO proteins minimizing possible structure anomalies when compared with overexpressed fluorophore-tagged proteins that may hinder the natural organization and interactions that form higher-order structures. For live-cell super resolution, we opted for a super-resolution optical fluctuation imaging (SOFI)-based method, enabling super resolution at low-intensity illuminations—orders of magnitude lower than comparable STORM methods—considerably reducing cell phototoxicity when compared with live dSTORM approaches, although providing a lesser resolution improvement (80–100 nm) and relying on fluorescent protein fusion constructs^26^.

We used total internal reflection fluorescence (TIRF)-based illumination in tandem with SRLM to super-resolve the immunolabelled NEMO nanoarchitecture near the surface of the cell membrane. This approach allowed us to observe the presence of large lattice structures in the sub-membrane region of U2OS cells (Fig. 1b). Consistent with a model of NEMO-IKK assembly predicted from IKK X-ray crystallographic data, we observed lattice-like structures composed of round clusters of NEMO of 50–100 nm, connected to each other by elongated bridges also composed of NEMO spanning over 400 nm in maximum diameter. These structures could not be accurately resolved using conventional methods including highly sensitive TIRF approaches as well as maximum intensity projections in widefield (Fig. 1b).

To ascertain whether these structures visible in fixed cells represented bona fide structures in live cells, we performed SOFI-based live super resolution using NEMO-defective cells^17^ transiently transfected with a GFP-NEMO fusion construct. Video acquisition shows that cells positive for green fluorescent protein (GFP) formed similar lattice structures (Supplementary Movie 1), to those observed with endogenous NEMO labelling in wild-type (WT) cells. In addition, SOFI high-speed acquisition indicated that these structures are highly dynamic.

We sought to confirm the presence of the lattice structure in primary cells. SRLM analysis revealed similar extensive lattice structures in human dermal fibroblasts (HDFs) approaching lengths of 1–3 μm (Fig. 1c). As with the U2OS cells, no distinguishable structures were observed in corresponding non-super-resolved TIRF or wide-field maximum intensity projection images (Fig. 1c).

To eliminate the possibility of a technical artefact, several control experiments were performed. An alternative antibody targeting NEMO revealed lattice structures of highly similar dimensions (Supplementary Fig. 1a,b). Potential artefacts due to nonspecific staining were excluded by performing SRLM on cells transfected with a small interfering RNA (siRNA) targeting NEMO and on cells immunolabelled with the secondary antibody alone. No structures of significant size were observed (Supplementary Fig. 1c,d). Further modifications to fixation and permeabilization showed no impact on the detected structures (Supplementary Fig. 1e,f). Finally, SRLM on a control protein, Calnexin, revealed no lattice-like structure (Supplementary Fig. 1g). These data, together with similar observations in live super-resolution microscopy convincingly indicated that the identified lattice structures are not a technical artefact.

### Inhibition of polyubiquitin chain binding abrogates lattices

In addition to the structural evidence of oligomerization of the IKK complex, further connections of oligomers may be facilitated by non-covalent binding via polyubiquitin chains. Several studies have revealed that the binding of K63 and Met1 (linear) polyubiquitin chains to two ubiquitin-binding domains, the NOA (also called UBAN) and ZF domains, located at the C-terminal extremity, are essential for NF-κB activation^27^,^28^,^29^,^30^,^31^,^32^,^33^,^34^ (see Fig. 1a). Although NEMO oligomerization and its binding properties to ubiquitin oligomers have been shown to be essential in canonical NF-κB signalling^11^,^12^,^35^,^36^, no report has yet demonstrated the extent to which these oligomers may be linked to each other via polyubiquitin chains to form a preformed higher-order structure (see Fig. 1a).

As polyubiquitin chains are constitutively present in the cell (reviewed in ref. 37) and NEMO contains ubiquitin-binding domains, we theorized that NEMO would be able to co-opt these polyubiquitin chains to facilitate the assembly of the lattice structure before cytokine induction. To test this hypothesis we performed SRLM on U2OS cells transfected with plasmids expressing the de-ubiquitinases (DUBs) CYLD, (cylandromatosis DUB) or OTULIN (ovarian tumour DUB with linear linkage specificity; also known as FAM105B). Although CYLD has been shown to hydrolyse both K63 and linear polyubiquitin chains^38^,^39^, the specificity of OTULIN is solely targeted towards linear polyubiquitin chains^40^.

**Figure 2:**
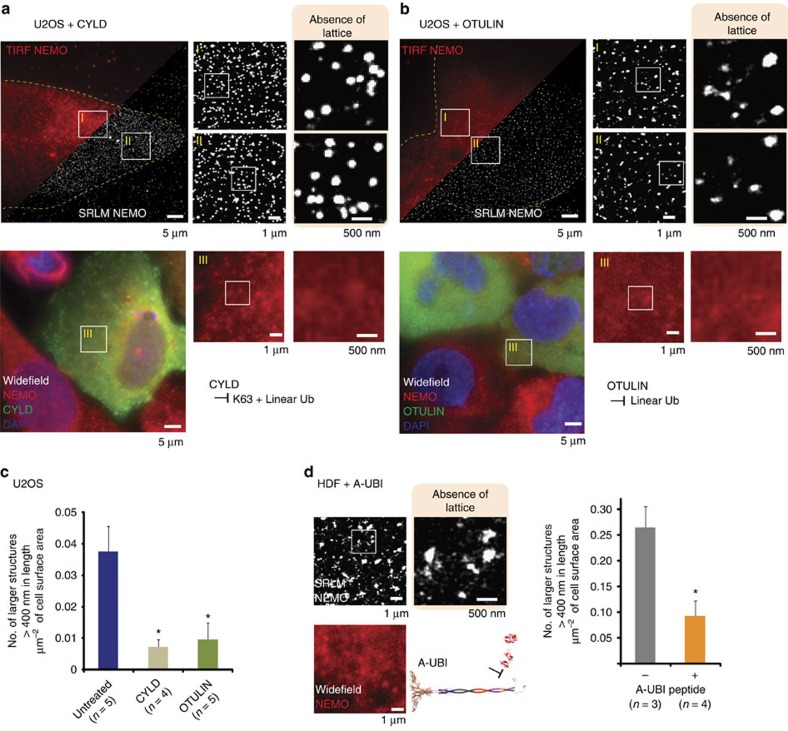
Higher-order NEMO lattice is dependent on K63 and linear polyubiquitin chain binding. U2OS cells overexpressing CYLD (**a**) or OTULIN (**b**) show no significant lattice structure of NEMO (upper micrographs). Lower micrographs show representative images of immunolabelled NEMO in cells transfected with DUB enzymes as shown by FLAG (green) immunolabelling. (**c**) Quantitative analysis of NEMO lattice size in U2OS cells. The number of large NEMO lattices was analysed in cells expressing DUB enzymes and compared with those in untreated U2OS cells. (**d**) Magnified images of SRLM and quantitative analysis on fibroblasts treated with the peptide A-UBI, which disrupts non-covalent binding between NEMO and polyubiquitin chains. Quantitative analysis measured number of NEMO structures over 400 nm in diameter per μm^2^ of cell area. *n* is the number of whole cells analysed per condition. Error bars are s.e.m. *P-value of <0.05 after a two-tailed *t*-test.

SRLM imaging of the CYLD-transfected cells revealed the removal of previously identified large lattice structures (defined as those over 400 nm in length, unless specified otherwise), although small clusters of NEMO were still apparent (Fig. 2a). As expected, we observed no structural alterations using conventional immunofluorescence at the same magnification (Fig. 2a, lower micrographs, insets) compared with untransfected cells. SRLM revealed a similar pattern of smaller structures in U2OS cells expressing OTULIN (Fig. 2b). These data strongly suggest that preformed higher-order NEMO structures are dependent both on linear and K63 polyubiquitin chains. This result was further validated by expressing CYLD in HDFs, which also revealed a similar reduction of large lattice structures coupled with an inability to translocate p65 to the nucleus following IL-1 stimulation (Supplementary Fig. 2).

Although every effort was made to remove bias in the observation of higher-order lattice structures between conditions, we sought to make our observations as quantitative as possible. We therefore developed an algorithm to perform unbiased analysis of the size of NEMO structures revealed by SRLM data (Methods; Fig. 2c). We compared the number of larger lattice structures (defined as those over 400 nm in length) per μm^2^ of cell surface area in untreated cells and those transiently transfected with DUBs. Quantitative analysis revealed the number of large lattice structures is reduced fourfold in U2OS cells transfected with DUBs, validating our observations.

The global catalytic removal of polyubiquitin chains by DUBs may have additional effects on cellular machinery and therefore contribute to an indirect loss of lattice structure. We therefore sought to specifically obstruct the binding of polyubiquitin chains to NEMO alone. The peptide A-UBI specifically blocks the binding of polyubiquitin chains to the NOA domain of NEMO^41^. Treatment of HDFs with A-UBI showed a loss of lattice structures via super-resolution imaging (Fig. 2d). Quantitative analysis revealed a threefold reduction in the prevalence of larger lattice structures. This was consistent with our hypothesis that polyubiquitin chain binding to the NOA domain was essential for the assembly of higher-order NEMO structures.

### Lattices provide a mechanism for proximity enhanced activation

**Figure 3:**
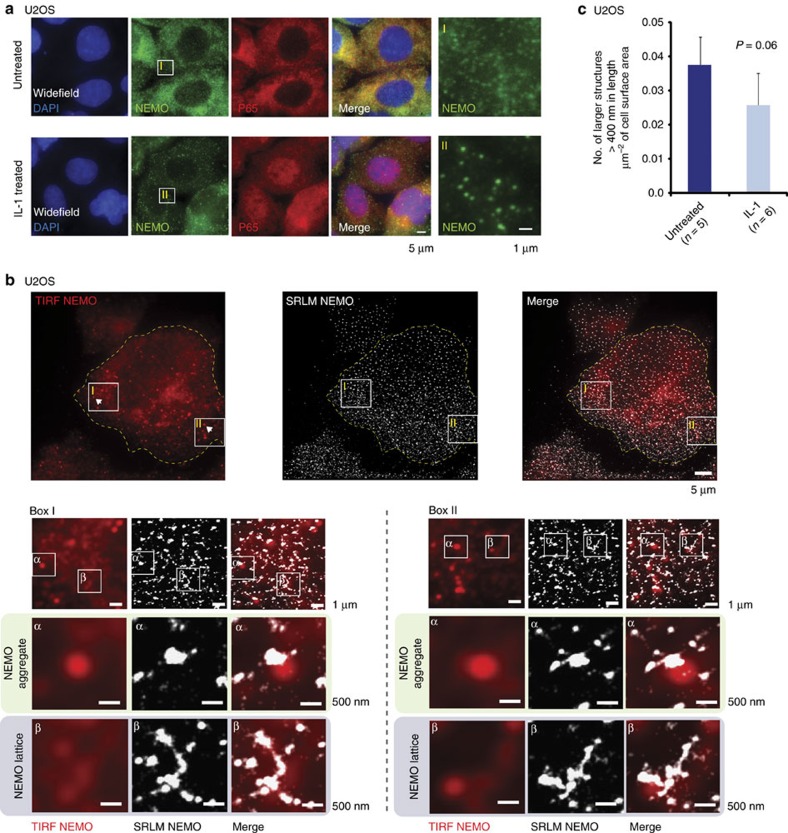
IL-1-induced NEMO aggregates are structurally distinct from preformed NEMO lattice in SRLM. (**a**) Conventional fluorescence imaging of U2OS cells shows formation of IL-1-induced NEMO aggregates concurrent with P65 translocation to the nucleus. Boxes I and II are magnified regions as indicated. (**b**) SRLM analysis can distinguish between IL-1-induced aggregates and the higher-order preformed NEMO lattice. Upper micrographs indicate a whole-cell image of NEMO immunolabelling. Box I and II are magnified sections indicated in the cell. Each box is further magnified to show IL-1-induced aggregates (α) in close proximity with preformed NEMO lattice structures (β). Whole-cell and magnified images show NEMO immunofluorescence from left to right in TIRF, SRLM and the corresponding merged image. (**c**) Quantitative analysis of NEMO lattice size in U2OS cells, comparing number of large NEMO structures in untreated and IL-1-treated cells. Quantitative analysis measured number of NEMO structures over 400 nm in diameter per μm^2^ of cell area. *n* is the number of whole cells analysed per condition. Error bars are s.e.m. *P-value of <0.05 after a two-tailed *t*-test.

It is well established that IL-1 induction of the NF-κB pathway requires the formation of a large signalosome at the receptor^42^. Indeed, we have recently demonstrated that these activating centres show recruitment of NEMO in the form of large aggregates^10^, which can be visualized in live cells using diffraction-limited spinning disk fluorescence microscopy (Fig. 3a). Therefore, to further investigate the functional significance of the preformed NEMO lattice, we stimulated U2OS cells with IL-1 and performed SRLM. On image reconstruction, we were able to distinguish between preformed lattice structures of NEMO and the more condensed IL-1-induced NEMO aggregates (Fig. 3b), the latter being absent in untreated cells (Fig. 1b). IL-1-induced aggregates were observed by TIRF and shown to be in the range of 400 nm in diameter after SRLM reconstruction. Magnified images shown in Box I and Box II show examples of both an IL-1 aggregate (α) and a preformed lattice (β) within the sub-membrane region. The IL-1 aggregates appear more dense, thus explaining their detection in conventional fluorescence imaging, whereas the preformed NEMO lattice is detectable only by SRLM. This may suggest a mechanism by which a preformed lattice compacts on itself to form a dense IL-1-induced NEMO structure, a consequence of cooperative activation of the high number of enzymatic complexes present in close proximity. Indeed, we did observe a slight reduction in the number of large lattice structures per μm^2^ following IL-1 induction (Fig. 3c).

### Catalytic IKK subunits are essential to lattice structure

**Figure 4:**
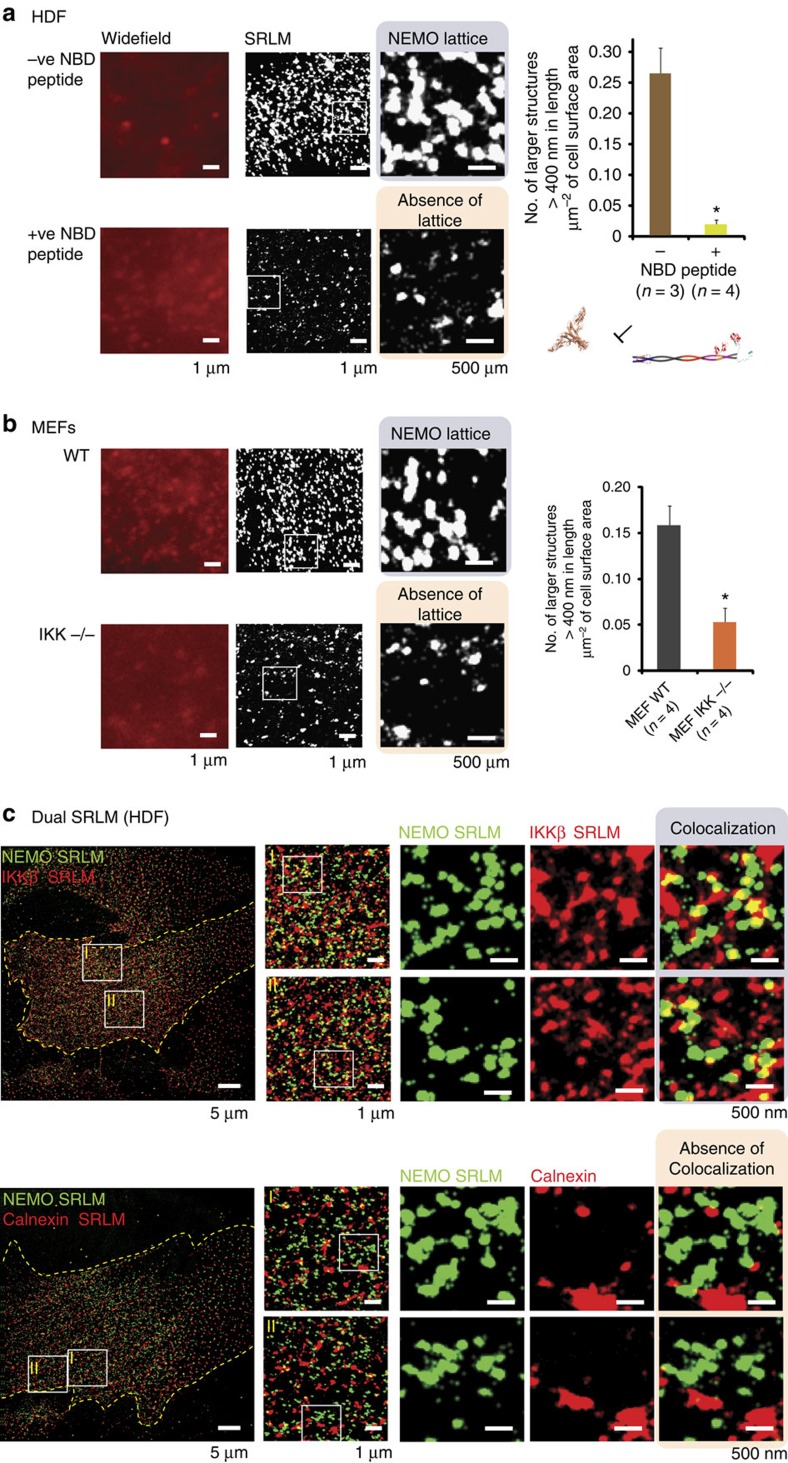
Catalytic IKK subunits are essential components of the NEMO lattice structure. (**a**) Disruption of NEMO lattice by abrogation of catalytic IKK subunit binding in WT HDFs. The peptide NDB disrupts NEMO lattice. Quantitative analysis of larger NEMO lattice structures show a significant decrease in cells treated with NBD. (**b**) Disruption of NEMO lattice in MEFs lacking the IKKα and β-subunits. Quantitative analysis of larger NEMO lattice structures show a significant decrease in MEF IKK^−/−^ cells. (**c**) Dual SRLM. NEMO lattices show colocalization with the catalytic IKKβ subunit in SRLM but not the cytoplasmic protein Calnexin. Quantitative analysis measured number of NEMO structures over 400 nm in diameter per μm^2^ of cell area. *n* is the number of whole cells analysed per condition. Error bars are s.e.m. *P-value of <0.05 after a two-tailed *t*-test.

Our previous work has shown that IL-1-induced NEMO aggregates visible using conventional microscopy colocalize with activated IKK^10^. Here we sought to investigate the degree to which the catalytic IKK subunits contribute to the formation of the preformed NEMO lattice. The Nemo-binding domain (NBD) peptide is a well-established inhibitor of the NF-κB pathway, which acts by preventing the interaction between NEMO and catalytic IKK subunits^43^. SRLM of HDFs treated with NBD peptide showed a dramatic reduction in the number of large NEMO lattices (Fig. 4a). Similarly, we saw a threefold reduction in preformed NEMO lattices in MEFs lacking IKKα and β-subunits (Fig. 4b). Interestingly, both inactive and active forms of the catalytic IKK subunits formed lattices resembling that of the NEMO structure identified using SRLM (Supplementary Fig. 3). This motivated us to perform dual STORM to observe whether the IKK subunits were co-localized. Unsurprisingly, preformed NEMO lattices appeared to co-localize with IKKβ but not the endoplasmic reticulum (ER)-associated protein, Calnexin (Fig. 4c). Together, these data strongly support an essential role for the IKKβ subunits in maintaining preformed NEMO lattices.

### Lattice structures are absent in IP-derived patient cells

To investigate the clinical relevance of the NEMO structures, we performed super-resolution analysis on fibroblasts with *NEMO* mutations acquired from samples with clinically defined IP. Fibroblasts from IP_GR_ contain a genomic rearrangement of exons 4–10 in *NEMO*, causative of the majority of IP cases^17^, resulting in a truncated protein^44^. Because of the concern of reduced binding sites available to the NEMO antibody in this mutant protein, we acquired fibroblasts from a second IP patient with a different disease-causing mutation in the *NEMO* gene. IP_SS_ cells contain a novel splice site mutation in *NEMO* resulting in the deletion of exons 4–6, and a truncated protein (Supplementary Fig. 4a and ref. 45). Significantly, the truncated version of NEMO in IP_SS_ cells retains the domains necessary to bind polyubiquitin chains, yet NEMO signalling is deficient in these patients. On the basis of structural modelling of the IP_SS_ NEMO dimer, we hypothesized that this mutation confers a different orientation of the dimer of NEMO UBD domains relative to the IKKβ amino-terminal binding site. This change in the spatial arrangement of UBDs is due to the modification of the left-handed superhelix of the coiled coil (Supplementary Fig. 4b). Importantly, the level of NEMO protein in cells from the IP_SS_ line is as high or higher than in WT control lines as measured by western blotting (Supplementary Fig. 4c).

**Figure 5:**
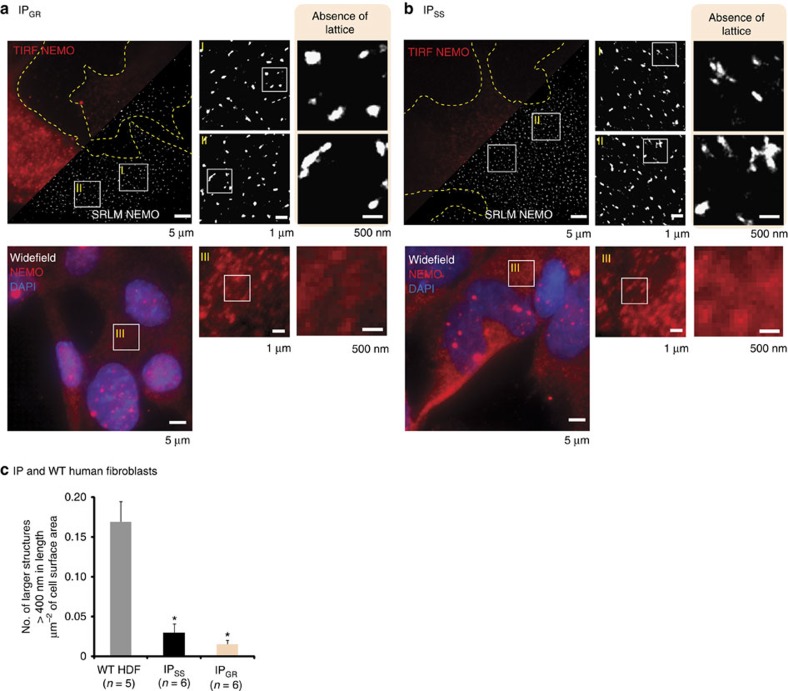
NEMO lattice structure is severely abrogated in IP-derived patient cells. SRLM analysis of NEMO immunofluorescence in fibroblasts from (**a**) IP_GR_- and (**b**) IP_SS_-derived samples show an almost complete inability to form higher-order NEMO lattice. (**c**) Quantitative analysis of NEMO lattice size in fibroblasts as measured by the number of NEMO structures over 400 nm in diameter per μm^2^ of cell area. *n* is the number of whole cells analysed per condition. Error bars are s.e.m. *P-value of <0.05 after a two-tailed *t*-test.

SRLM analysis on both these cell lines revealed the presence of small clusters of NEMO with no significant higher-order lattice structure observed (Fig. 5a,b) when compared with that of WT HDFs. Indeed, we observed an almost complete absence of preformed lattice structures over 1 μm in length (uniquely observed in HDFs compared with other cell types). Quantitative analysis confirmed our observation showing a substantial decrease in the frequency of large NEMO lattices in IP derived cells compared with their WT counterparts (Fig. 5c).

Apart from the reduced immunolabelling in IP_GR_-derived cells (expected from truncated proteins and thus reduced antibody binding), neither conventional immunofluorescence nor TIRF microscopy revealed any differences in NEMO staining (Fig. 5a,b). Indeed, the only structural difference observed between WT HDFs and IP-derived fibroblasts identifiable using conventional fluorescent microscopy was that of an inability of the IP-derived cells to form IL-1-induced NEMO aggregates (Supplementary Fig. 5).

### Quantitative analysis of NEMO lattice structures

Although we had provided some quantification of our SRLM data, we sought to further interrogate our algorithm data. We therefore assessed the prevalence of the larger NEMO lattices relative to the total number of structures in each cell type or condition, as well as the density of each structure in IL-1-stimulated cells.

**Figure 6:**
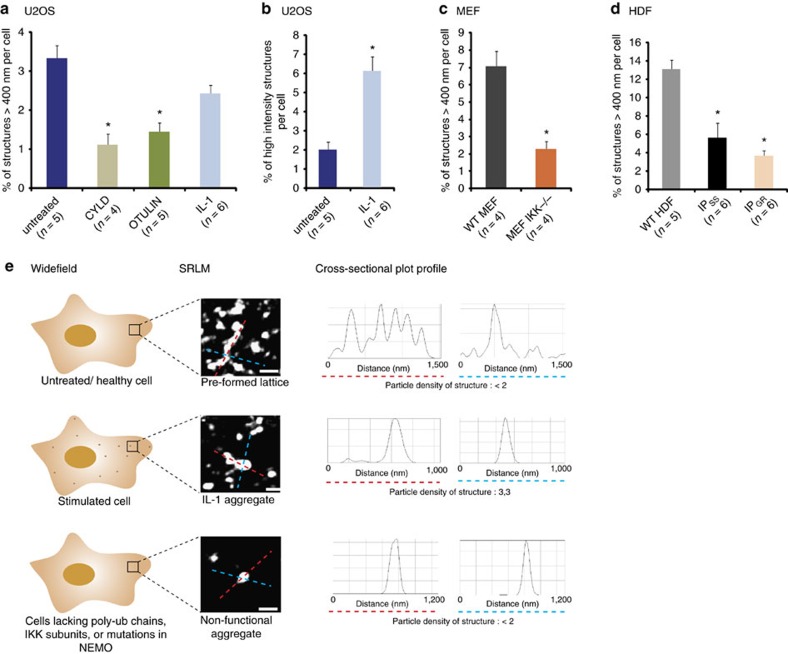
Quantitative analysis and characterization of NEMO higher-order structures by SRLM. Quantitative analysis of the length or intensity of NEMO structures over 400 nm in length as a percentage of total NEMO structures across different cell types and conditions in (**a**,**b**) U2OS cells, (**c**) MEFs and (**d**) fibroblasts. Quantification of size (**a**,**c**,**d**) measured those structures over 400 nm in length, whereas quantification of intensity (**b**) was measured as those structures with over two particle detections per nm^2^. (**e**) Schematic characterization of distinguishing structures in different cellular conditions. In untreated or healthy cell types, only SRLM reveals extensive NEMO lattice structures shown with an accompanying cross-sectional plot profiles and associated particle density as measured by metric analysis. In stimulated cells, conventional microscopy reveals punctate NEMO aggregates, which can be distinguished from preformed lattice structures in SRLM. Cells unable to bind catalytic IKK subunits and polyubiquitin chains cannot form extensive lattice structures, nor high-density structures. Each representative image is associated with its plot profile, indicating it's maximum length on the *x* axis as shown by a dashed red or blue line across the structure in question. Scale bar, 500 nm. *n* is the number of whole cells analysed per condition. Error bars are s.e.m. *P-value of <0.05 after a two-tailed *t*-test.

Analysis of the maximum diameter of NEMO structures in U2OS cells across multiple conditions revealed smaller structures of 50–300 nm to be the predominant species (Supplementary Fig. 6a). Large lattice structures represented only 3.3% of the total number of NEMO structures in untreated U2OS cells (Fig. 6a). In contrast, cells treated with CYLD and OTULIN were revealed to have only 1.1 and 1.5% of NEMO lattices over 400 nm. This represents a three- and twofold decrease, respectively. Interestingly, we observed that the fold decrease in structures was more pronounced when comparing the largest higher-order order structures over 550 nm between conditions. In restricting our analysis to this size bin, we observed a ten- and fourfold decrease of CYLD and OTULIN-treated cells respectively compared with controls (Supplementary Fig. 6a). This reduction was enhanced in CYLD-transfected cells compared with those transfected with OTULIN. Indeed, overexpression of CYLD led to a significant increase in the percentage of smaller structures of 20–50 nm, possibly representing NEMO reduced to its basic dimer unit. The more severe effect by CYLD is likely to be due to the combined elimination of K63 and linear chains, whereby K63 linkage represents the predominant species. Abrogation of polyubiquitin chains should reduce the lattice to its multimeric IKK/NEMO complex size of 128 nm. Although we did not observe a significant increase in lattices in the range of 100–300 nm in DUB-treated cells, this could well be explained by the transient nature of the experiment, whereby only the largest structures have been affected after 48 h. Importantly, the transient expression of either CYLD or OTULIN lead to the functional disruption of the NF-κB pathway. Thus, it appears that although large preformed higher-order lattice structures represent only a small proportion of total structures in the cell, they provide sufficient and essential higher-order platforms for a functional signal transduction response.

Analysis of NEMO structures in IL-1-stimulated cells compared with untreated cells revealed a small but general reduction in larger lattice structures, although this only achieved significance for a single bin size (500–550 nm; Supplementary Fig. 6a and Fig. 6a). Given IL-1-induced NEMO aggregates can be observed with conventional microscopy (Fig. 3), we asked whether they were composed of a higher density of NEMO particles. Quantitative analysis revealed that there was no significant difference in the predominant species of low-density structures (<2 particle detections per nm^2^; Supplementary Fig. 6b). In contrast, high-intensity structures increased from 2 to 6% in IL-1-treated cells (Fig. 6b). This threefold increase was more pronounced in comparisons of higher particle density (Supplementary Fig. 6b). Taken together, the modest reduction in large structures concurrent with the appearance of IL-1-induced NEMO aggregates may suggest a structural rearrangement of preformed lattices into IL-1 activation centres. Furthermore, the increased density of NEMO structures in IL-1-stimulated cells may lend further support to a model of proximity enhanced activation and cooperativity.

Having identified that transient expression of DUBs could significantly decrease the proportion of preformed NEMO lattices, we asked whether cells lacking catalytic IKK subunits showed a similar reduction. We found a threefold decrease in lattice prevalence in MEF IKK^−/−^ cells (Fig. 6c). Interestingly, this corresponded with a robust and significant increase in base unit NEMO dimers (<100 nm) in IKK^−/−^ cells (Supplementary Fig. 6c). Alhough we did not observe a similar increase in the prevalence of smaller structures post DUB treatment, this is probably explained by the prolonged absence of IKK subunits in these stable knockout cell lines rather than a transient removal of polyubiquitin chains.

Lastly, we compared the maximum diameter of structures from WT HDFs and IP-derived cells (Fig. 6d, expanded data in Supplementary Fig. 6d). Our analysis validated our observations of a significant reduction in preformed lattice structures in IP-derived cells, with a two- to threefold decrease in the prevalence of large structures compared with control cells. This effect was exacerbated in the more severe IP_GR_ patient cells. As fibroblasts exhibit larger structures than U2OS cells, we further compared even larger higher-order lattices over 800 nm in length (Supplementary Fig. 6d). These structures represented 0.6% of all structures in the cell, but only 0.05% in IP_SS_ cells. We observed no structures of this size in IP_GR_ cells. Although these numbers represent a small proportion of the total structures, those over 400 nm in size occupy over 50% of total NEMO structure area in WT HDFs, but only 20 and 13% in IP_SS_ and IP_GR_ cells, respectively (Supplementary Fig. 6e). We further observed a significant increase in the prevalence of smaller structures of 200–300 nm in IP-derived cells, reinforcing the notion that these cells are unable to form NEMO lattices of significant size, but yield an increased proportion of NEMO conglomerates of a non-functional nature. The shift in the distribution of structure size was more severe in IP_GR_ cells, although this is likely to be due to the more severe nature of the mutation. Although the increased proportion of small structures in IP patient cells within the range of 200–300 nm does not directly equate to the NEMO dimer ‘base unit', these structures may represent non-functional conglomerates of ‘sticky' NEMO protein. Although they are still able to bind IKK subunits, this alone cannot facilitate significant lattice formation without polyubiquitin chains.

The combination of these data are summarized in the schematic representation in Fig. 6e. Under normal conditions, unstimulated cells contain preformed lattices extending from 400 nm to over 1 μm in length held together by delicate bridges <100 nm in width. Importantly, cells stimulated with IL-1 largely maintain these lattices but reveal highly dense smaller NEMO aggregates 200–400 nm in size, which can be visualized by conventional microscopy. In contrast, cells in which the interactions of NEMO with polyubiquitin chains or catalytic IKK subunits are perturbed form small non-functional NEMO aggregates. Importantly, the presence or absence of preformed lattices is only distinguishable by SRLM.

## Discussion

In this report we use SRLM to demonstrate the existence and, indeed, extent of a preformed higher-order lattice structure of NEMO, as well as its functional significance in the context of a genetic disease (Fig. 6). Although such a higher-order structure has been hypothesized and inferred from biochemical data, to the best of our knowledge this is the first time it has been visualized in both live-cell and fixed-cell conditions, and therefore the strongest evidence that exist to date.

Despite the size of the NEMO higher-order lattice surpassing the diffraction limit of light, the delicate NEMO bridges along which it is connected appear to be within the range of 20–100 nm (cell type dependent), thus obscuring their observation using diffraction-limited microscopy. Alternative techniques have been used to reveal similar nanoscale structures, including electron microscopy and PALM. However, to preserve the structural integrity of the delicate lattice within the context of endogenous NEMO expression levels, we believed a dSTORM strategy was best suited to this application, although a SOFI-based method was also used to demonstrate that the lattices could still be observed in live-cell conditions.

Notably, our results contribute significantly to the mechanistic understanding of how a regulatory protein can so efficiently and exquisitely react to external stimuli. We determined that a small number of preformed open-ended NEMO lattice structures linked by catalytic IKK subunits and polyubiquitin chain binding are sufficient to rapidly activate the NF-κB cascade. Although it has been shown that polyubiquitin chains bind NEMO on IL-1 stimulation^46^, our data strongly suggest both K63 and linear chains are necessary and exist within a preformed NEMO structure. An explanation for this inconsistency between our and previous experimental data may lie in the fact that this delicate non-covalent association of reticulated NEMO may be destroyed by insensitive biochemical techniques. Interestingly, the proportion of preformed structures varied across different cell types, although this may be attributed to the well-documented variation in the concentration of polyubiquitin chains between cell types^47^. We observed that the appearance of a few highly dense NEMO aggregates in IL-1-stimulated cells correlated with a very small reduction in open-ended NEMO lattices. Although we cannot exclude additional protein factors, the data presented here supports a mechanism by which a small number of lattices are primed to swiftly respond to external stimuli and ‘fold-in' on each other to create compact NEMO-IKK signalosomes leading to a proximity-enhanced response. Such a mechanism would require a highly dynamic structure. Both our live-cell data and a previous report^20^ provide strong experimental evidence to show the highly dynamic nature of NEMO oligomers. Indeed, this may further explain the modest reduction (Fig. 6a) in open-ended lattices in IL-1-treated cells, in which we would expect to observe some latency in the reformation of the lattice. Instead, a rapid and highly dynamic feedback mechanism is implicated in the reformation of the NEMO lattice structure on IL-1 induction. It would therefore be highly valuable to further investigate the nature of this dynamic interaction between inactive and activated cellular states with the advancement of such technologies in a broader study. In particular, NEMO ubiquitination—which corresponds to a covalent attachment of non-degradative polyubiquitin chains (linear and K63) to NEMO—could considerably enhance the conversion of preformed open-ended NEMO lattice structures into activating centres, where specific ubiquitin ligases are presumably present. Importantly, the small proportion of higher-order NEMO lattices and subsequent IL-1-induced signalosomes further provides a mechanism by which the signalling response could ‘ignore' biological noise. This property, in conjunction with an ability to mediate proximity-enhanced activation, would effectively facilitate a binary biological switch.

The importance of such a higher-order lattice of NEMO is apparent from our data showing the near elimination of these structures in IP-derived cells. At first glance, the NEMO mutation in IP_SS_ cells confers no apparent detriment to the function of the protein, apart from a reduction in length. Importantly, the domains necessary for IKKβ and polyubiquitin chain binding are still present in these cells. Thus, NEMO should be able to facilitate activation of the signalling cascade. Yet, our analysis of structural data (Supplementary Fig. 4) indicates that the IP_SS_ mutation results in the ubiquitin-binding domains being misconfigured, leading to incorrectly positioned polyubiquitin chains, obstructing their ability to form a higher-order lattice structure. Therefore, in demonstrating that cells with this mutation lack higher-order structures before stimulation, we show the extent to which such accurate structural oligomerization and cooperativity is required for NF-κB signalling. Indeed, our data provide evidence that despite the broad spectrum of IP causing mutations, the common molecular cause for disease may be an inability to form higher-order lattices of significant size and number. These structures permit the signal transduction cascade through cooperativity, to achieve rapid Hill function responses once the appropriate threshold is achieved.

Higher-order structures constitute a very small proportion of total structure number, yet are absent in IP patient-derived cells, which lack a functional NF-kB response. This suggests that only a small number of large higher-order structures are necessary to efficiently regulate reliable signal transmission and interpretation by balancing signal intensity, while acting as a noise filtering mechanism. This concept bolsters previous observations where it has been shown that NF-κB signalling is activated in a digital manner, rapidly reaching a stimulus saturation point, exemplified in rapid Hill function responses^48^.

Although there exist simple methods for diagnosis of IP, the demonstration here that IP patients harbouring different mutations have a common molecular defect in the higher-order structure of NEMO protein may bypass the mutational screening and characterization required for identifying NEMO mutations. It is possible to speculate that this method may be useful for prenatal diagnosis in families in which IP is suspected but not fully confirmed in males but also, as evident by our study, in females in which skewed X-inactivation has not occurred. In our analysis we were able to show a statistical difference with only six cells from different clinical samples, suggesting the isolation and limited expansion of amniocytes may be useful in augmenting such a diagnosis. The expansion of such super-resolution techniques may therefore provide not only invaluable mechanistic insight into disease but also facilitate the genetic diagnosis of a clinically severe disorder.

## Methods

### Structural modelling of NEMO

A modelling of the human dimeric NEMO protein (residues 49–419) with K63 di-Ub chains was undertaken based on COILS^49^ and PAIRCOIL2 (ref. 50) coiled-coil prediction algorithms and the following crystal and NMR structures of NEMO fragments: kinase-binding domain (PDB ID: 3BRV, residues 49–109); Helical domain 2 (HLX2, PBD ID: 3CL3, residues 193–249), coiled-coil domain 2–leuzine zipper (CC2-LZ, PDB ID: 2V4H and 4BWN, residues 257–344) and ZF (PDB ID: 2JVX, residues 392–419). All regular heptad phases (abcdefg)_*n*_ within the predicted fragment of NEMO (residues 49–364), as well as coiled-coil discontinuities such as stutters (deletion of three residues) and stammers (deletion of four residues), were correctly predicted using PAIRCOIL2 when compared with the heptad phases identified from available crystal structures by the coiled-coil analysis programme SOCKET^51^. This includes, for instance, one stutter and one stammer at positions 103 and 295, respectively. Two other highly predictable discontinuities at position 203 (stutter) and 256 (deletion of two residues) were taken into account to accommodate the heptad phases at the KD/HLX2 and HLX2/CC2-LZ junctions. A first model was built using O^52^ by assembly of crystal and NMR NEMO fragments with the following modelled regions: 110–192 (CC1, 82 aa), 250–256 (7 aa), 345–364 (20 aa) and 365–391 (27 aa). The long WT CC1 (110–192) and the short mutant CC1 (110–133) fragments were generated using CCBuilder^53^ with a radius of 5.07 Å, a superhelical pitch of 225.8 Å and an interface angle of 26.42°. The fragment building corresponding to the disordered region between CC2-LZ and ZF domains (residues 365–391) was based on the circular dichroism (CD) spectra analyses of the 248–344, 248–419 and 392–419 constructs of human NEMO (CD spectra available upon request). This disordered fragment was manually generated and refined from initial atomic coordinates derived from a I-TASSER-generated model based on threading algorithm^53^. The NEMO:IKKβ:K63-di-Ub complexes (WT and Mu) were built by homology modelling using as structural templates the crystal structure of the mouse CC2-LZ in complex with a K63 di-Ub chain (PDB ID: 3JSV^54^) or the modelled NEMO ZF in complex with a K63 di-Ub chain^55^. This structural modelling of NEMO is consistent with a recent *in vitro* EPR study showing that NEMO forms a parallel coiled-coil dimer in solution^20^. The incomplete crystal structure of the human IKKβ (residues 16–665, PDB ID:4E3C) and its short C-terminal fragment bound to the NEMO N terminus (NDB, residues S705–Q743, PDB ID:3BRV) were previously described^15^,^56^. PyMOL (version 1.7, Schrödinger) was used for molecular display and structural figures.

### Expression constructs and antibodies

The CYLD-FLAG expression construct has been previously described^38^ (kind gift of Dr Gilles Courtois), whereas the OTULIN-GFP expression construct was obtained from OriGene Technologies (USA, catalogue number RG224840). The siRNA targeting NEMO was acquired from Sigma. Primary antibodies for labelling NEMO for SRLM were obtained from Santa Cruz (FL-419; polyclonal) and BD Transduction Laboratories (611306; monolconal). The latter binds to amino acids 278–396. The anti-P65 antibodies were obtained from Santa Cruz (sc-109; sc-8008), whereas the anti-FLAG antibody was purchased from Sigma (F1804). The catalytic IKK subunit antibodies used for SRLM were as follows: Ab6146 Rabbit polyclonal to IKKβ (Abcam), Anti hPhospho IKK alpha S176/S180 catalogue number MAB3768 (R&D), Ab194528 Rabbit polyclonal to IKKα and IKKβ (phospho S176 & S177) (Abcam).

The following secondary antibodies were used: anti-rabbit AlexaFluor 488 (A11008, Invitrogen), anti-mouse AlexaFluor 488 (A-11001, Invitrogen), anti-rabbit Atto 550 (611-154-122S, Rockland), anti-rabbit Atto 647N (611-158-122S, Rockland), anti-rabbit AlexaFluor 647 (A-31573, Invitrogen) and anti-mouse AlexaFluor 647 (A-21236, Invitrogen). We observed as others have that the best SRLM acquisitions were acquired with a 647 dye. We noted that the monoclonal antibody may reveal less extensive structures, as it binds to the UBAN domain of NEMO, possibly excluding the population of NEMO forming extensive lattice structures. Therefore, all metric analysis was performed using the polyclonal antibody.

### Cell culture and transfection conditions

All patient material was gathered, after receiving informed consent from participants, under protocols approved by the Declaration of Helsinki, which was approved by the ethics committee at Hôpital Necker. U2OS cells and WT fibroblasts (WT HDFs) were obtained from Lonza (USA). WT MEFS were obtained from ATCC (USA). MEF IKK^−/−^ cells were a kind gift of I.M. Verma (The Salk Institute Institute for Biological studies, La Jolla, CA). All cells were grown in complete RPMI medium (Gibco) containing 10% fetal bovine serum (PAA) and were screened for mycoplasma before experimental use. Cells were authenticated by western blotting where appropriate. Plasmids and siRNA were transfected into U2OS cells using Fugene HD (Roche) and into fibroblasts using the Invitrogen Neon Transfection system. Live-cell experiments were performed as previously described^10^ using IP_GR_ NEMO defective cells. Transiently transfected cells were imaged 48 h after transfection. U2OS cells and fibroblasts were stimulated with 10 ng ml^−1^ IL-1β (H2691, Sigma) for 5 and 15 min, respectively, before fixation and imaging. The peptides A-UBI (catalogue number 481418, Calbiochem) and NBD (480025, Calbiochem) were used at a final concentration of 20 μM on WT HDFs. Cells were incubated with aforementioned peptides for 2 h, followed by treatment with IL-1, before fixation.

### Western blottings

Cells (10^6^) were lysed in 200 μl of lysis buffer. After centrifugation at 10,000 *g*. Ten microlitres of supernatant corresponding to 10^5^ cells were loaded onto a 12% SDS–PAGE, followed by western blotting using a monoclonal antibody against NEMO. The human nucleoside diphosphate kinase B was used as a loading control.

### Sample preparation for SRLM

Glass coverslips were washed three times in optical grade acetone, methanol and deionized water. Coverslips were then sonicated in 0.1 M KOH for 45 min, rinsed and further sonicated in deionized water. Cells were resuspended in 100% ethanol and exposed to ultraviolet radiation. Cells were fixed with 3.7% stabilized formaldehyde for 10 min at room temperature and then permeabilized with 70% ethanol. After blocking with 1% BSA-Tween for 30 min, the samples were incubated with primary antibodies at 4 °C overnight, washed three times with 1% BSA and incubated with secondary antibodies for 1 h at room temperature. To maximize the number of detected molecules, care was taken to minimize photobleaching: cells were embedded in oxygen scavenger buffer as previously described^25^, imaged on the same day of labelling and kept in the dark until imaged. Tetraspeck beads (200 nm diameter; Invitrogen) were mounted with the sample as fiduciary landmarks and later tracked for computational drift correction.

### Image acquisition

Samples were imaged on a custom-built PALM/STORM microscope as described before^57^. For each coverslip, preselected fields of view were chosen typically containing one cell. We developed the automated acquisition sequence based on the open-source Micro-Manager microscopy acquisition software^58^. First, the field of view was centred to include as much of the cytosol as possible, to maximize the area acquired over the flatter cell membrane. Cells were then imaged by TIRF illumination with a 488 nm, 568 nm and/ or 647 nm laser. We then performed a SRLM image-stream acquisition for the 647 channel (635 nm laser-excitation at 1.7 kW cm^−2^, 662–690 nm emission) or the 568 channel (561 nm laser excitation at 2.4 kW cm^−2^, 589–625 nm emission), each composed of 20,000 images acquired at 33 ms intervals. Dual dSTORM images were generated by first performing an acquisition as described above in the 647 channel, followed by the 568 channel. Imaging parameters were set using Micro-Manager running on a desktop PC. Laser control was achieved with custom software^57^. Before particle detection and localization, fiduciary beads were identified and marked, typically 1–5. Sample drift was then calculated by tracking the group displacement of selected beads throughout the acquired image sequence. After particle detection and localization, the position of each particle was readjusted by subtracting the drift identified at the corresponding time point.

The diffraction limited *z*-stack acquisition was performed at 300 nm *z*-steps encompassing the size of the cells. This was performed to determine the proper localization and expression levels of the proteins of interest in the analysed cells.

### Live-cell image acquisition

Imaging of NEMO-GFP-expressing cells was performed using an N-STORM inverted microscope (Nikon) in TIRF mode. A 488 nm excitation laser was angled through the back focal plane of an original magnification × 100 TIRF objective (Nikon). A range of laser powers were tested for imaging the samples; as GFP intensity fluctuations are minimal regardless of laser power in the absence of a specialized imaging buffer, similar SOFI-based reconstructions were obtained at all powers tested. As a result, the live-cell movie displayed was obtained at 0.083 kW cm^−2^. Emitted signal from the excited GFP molecules was collected by an electron-multiplying charge-coupled device camera (iXon Ultra 897, Andor), with a pixel size of 160 nm.

### Fixed-cell super-resolution image reconstruction

Final super-resolution reconstructions were then generated in Fiji^59^ through the QuickPALM algorithm^57^ by creating images with 20 nm pixel size and additively superimposing a Gaussian kernel of 20 nm full-width half-maximum to each particle.

### Live-cell super-resolution image reconstruction

Final super-resolution reconstructions were generated in Fiji^59^ through a custom-made SOFI-based algorithm using fourth-order SOFI and generating images with 40 nm pixel size. Super-resolution Movie 1 analysed through the custom NanoJ-SRRF V1.0 ImageJ plugin is available upon request to the authors.

### Quantitative analysis

Protein cluster segmentation was performed through a dedicated algorithm implemented in Jython, running within the Fiji software^59^. Here the algorithm uses a Huang threshold method^60^ to segment clustered particle detections, this approach showed optimal performance in the identification of NEMO particle clusters. Background detections (false detection) caused by noise fluctuations or motile unbound labelled antibodies were minimally present in the thresholded images. For each segmented particle cluster in the rendered image, the following properties were measured: particles density, area, circularity and diameter. We verified that cell illumination was considerably homogenous for the individual cell regions-of-interest, but slightly non-homogeneous for the full field of view due to the Gaussian profile of the laser illumination in our microscopy setup. In all graphs, *n* is the number of whole cells analysed per condition.

### Statistical analyses

We used StatPlus to create histogram bin sets for the quantification of comparisons between cell types and treatments. The error bars are representative of s.e.m. Statistical analyses were performed using a two-tailed Student's *t*-test, to determine statistical significance that is expressed as *P<0.05. Data shown are that of biological replicates from various experiments performed multiple times.

### Data availability

The data that support the findings of this study are available from the corresponding authors upon request.

## Additional information

**How to cite this article:** Scholefield, J. *et al*. Super-resolution microscopy reveals a preformed NEMO lattice structure that is collapsed in incontinentia pigmenti. *Nat. Commun.* 7:12629 doi: 10.1038/ncomms12629 (2016).

## Supplementary Material

Supplementary Information Supplementary Figures 1-6

Supplementary Movie 1 Live-cell imaging of NEMO-GFP transfected IP_GR_ cell. Left panel, TIRF image acquisition, scale bar 5 μm; Middle panel, 4th-order SOFI super-resolution reconstructions, scale bar 5 μm; Right panel, insets of middle panel, scale bar 1 μm.
