# Supplementary material for: Super-resolution microscopy reveals a preformed NEMO lattice structure that is collapsed in incontinentia pigmenti
Source: Nat Commun. 2016 Sep 2;7:12629. doi: 10.1038/ncomms12629 (PMC5025789; doi:10.1038/ncomms12629)
Supplement: Supplementary Information — Supplementary Figures 1-6 [file ncomms12629-s1.pdf]

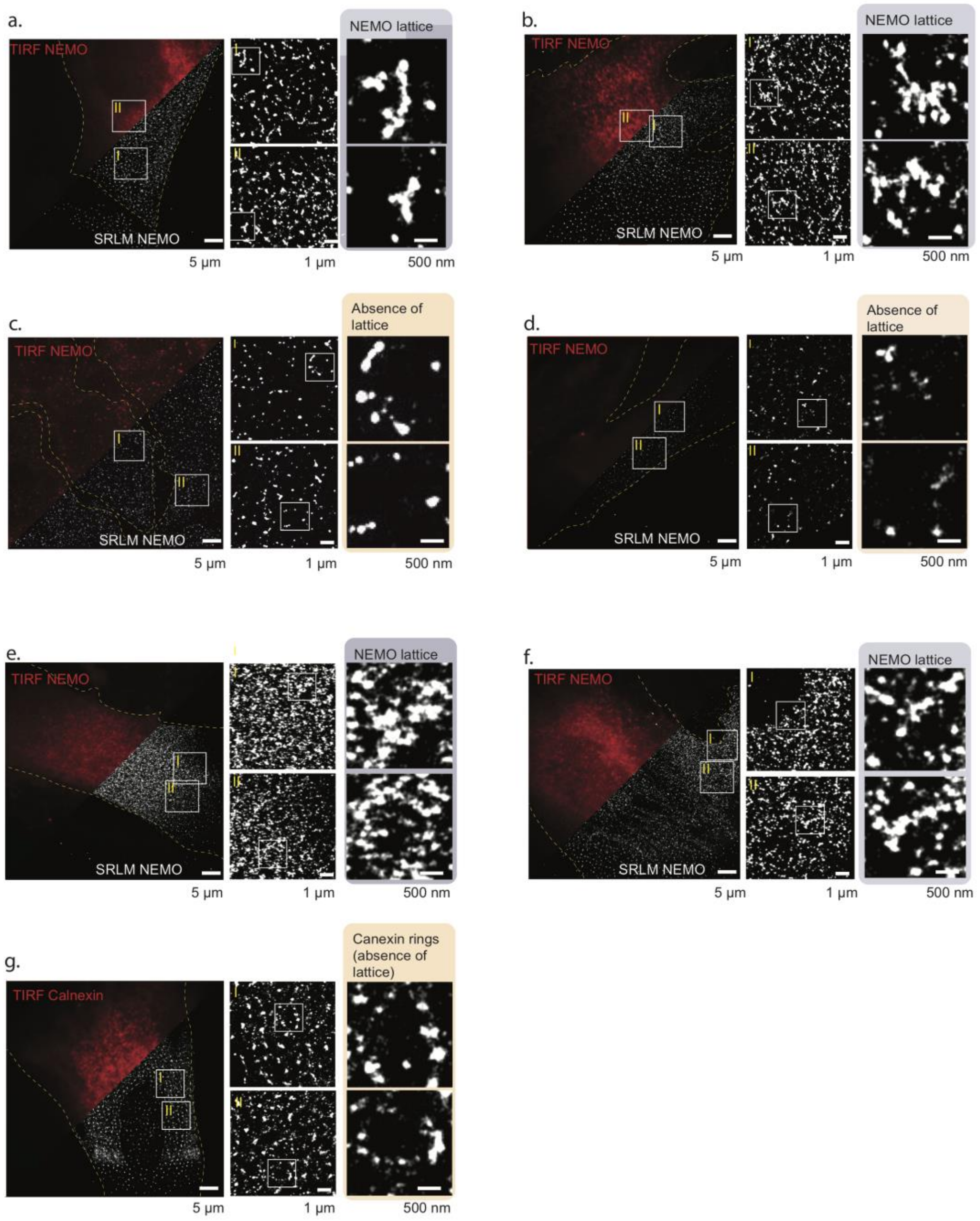

Supplementary Figure 1 Confirmation of lattice structure specificity.

SRLM of NEMO in U2OS cells with a polyclonal antibody (a) reveals highly similar extensive branching of NEMO lattice to that of the monoclonal antibody (b) in WT HDFs. In contrast, SRLM analysis of U2OS cells treated with siRNA targeting NEMO (c), as well as samples prepared without a primary antibody (d) showed an absence of lattice structures indicating the specificity of the immunofluorescence staining. SRLM of NEMO with different fixation techniques (e; modified by using paraformaldehyde and a quenching step) and alternative permeabilisation methods (f; saponin) does not alter the presence of the lattice structures. g) SRLM of an ER-associated cytoplasmic protein, Calnexin, forms distinct structures adapting the ring-like formation and not a NEMO-like lattice.

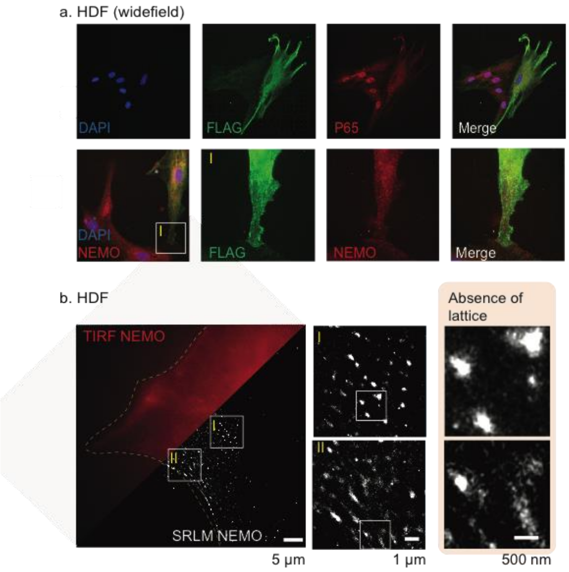

Supplementary Figure 2 Higher-order NEMO lattice is dependent on K63 and linear polyubiquitination in primary cells.

a) Upper micrographs show cells over-expressing CYLD cannot translocate P65 in response to IL-1 stimulation. Lower micrographs show identification of a transfected cell in close proximity to an untransfected cell; FLAG signal can be specifically identified in the magnified image of Box I showing definitive FLAG expression. The same cell as indicated by * was analysed with SRLM (b) and shows no significant NEMO lattice structure.

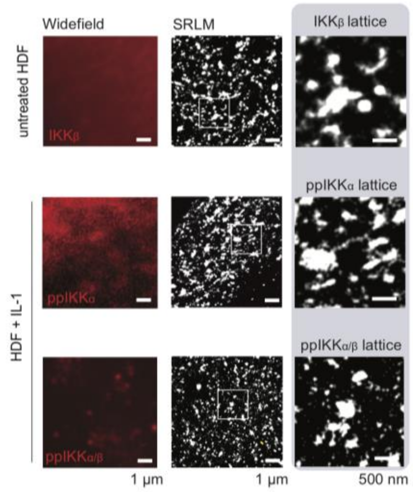

Supplementary Figure 3 Catalytic subunits can form similar lattice-like structures

Inactive IKKβ, and activated forms of IKKα and β form branched lattice-like structures after performing SRLM.

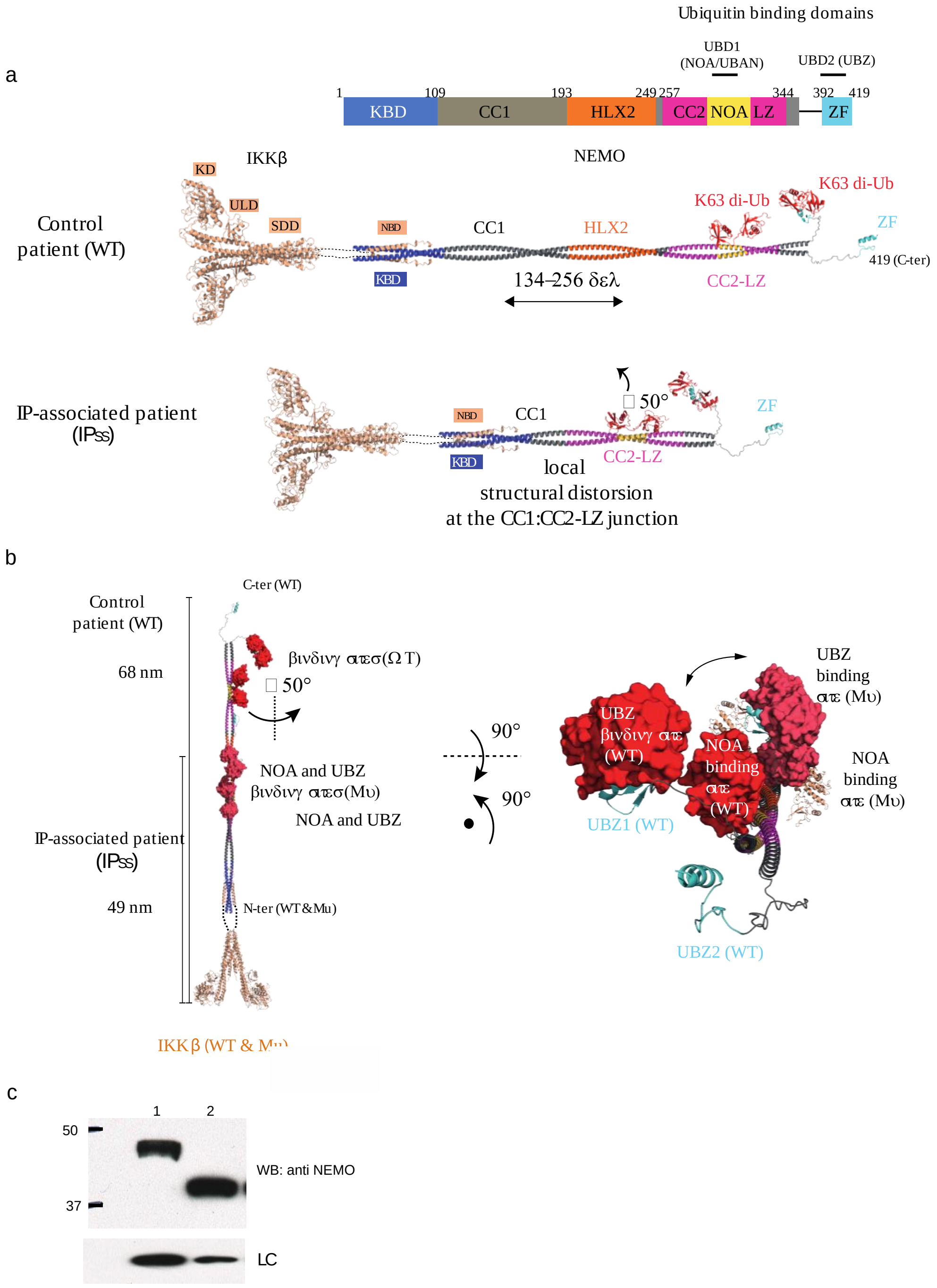

Supplementary Figure 4 Characterisations of the models of the WT and mutant NEMO derived from IP_{ss}

a) The IP-associated deletion mutant (134-256 del) contains all essential domains required for producing a functional NEMO protein. This deletion which introduces a novel junction between

Q133 (CC1) and G257 (CC2-LZ) leads to a discontinuity in the regular heptad repeats of CC1 and CC2-LZ domains and give rise to a local structural distortion of the a-helix and modification of alpha-helix packing, which may alter NEMO oligomerization. b) Two different views of the structure superposition among the WT and mutant. Structures were superimposed using the α Carbon of N-terminal kinase domain (KD). K63 di-Ub chains bound to NOA or ZF sites were depicted in surface representation for the WT (bright red) and the mutant (red blade). In addition to length shortening, the deletion of 123 residues in the mutant (residues 134-256), likely induces a modification of the left-handed superhelix of the NEMO coiled coil structure. This leads to different orientations of NOA and ZF UBDs relative to the IKKβ binding site as indicated. These differences in the spatial arrangement of UBDs may also contribute to impairment of the formation of a lattice structure in the cell. For clarity, only the dimeric NEMO-IKKβ complex with a binding stoichiometry of one di-Ub chain per UBD (NOA or UBZ) is shown. c) Western blot targeting NEMO showing similar levels of expression in WT (1) vs IP_{ss} (2) cell lines. LC; Loading control.

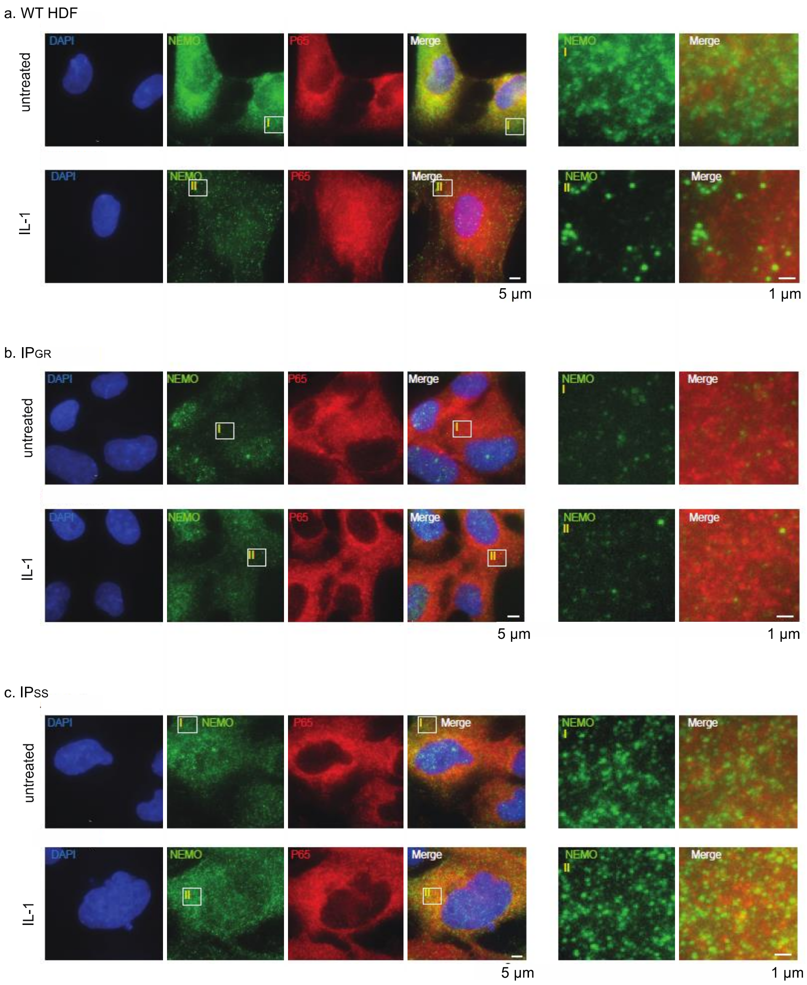

Supplementary Figure 5 IP patient fibroblasts cannot form IL-1-induced NEMO aggregates. Wild-type primary fibroblasts (a) but neither IP_{GR} (b) nor IP_{SS} (c) cells form IL-1-induced NEMO aggregates visible by conventional fluorescent microscopy.

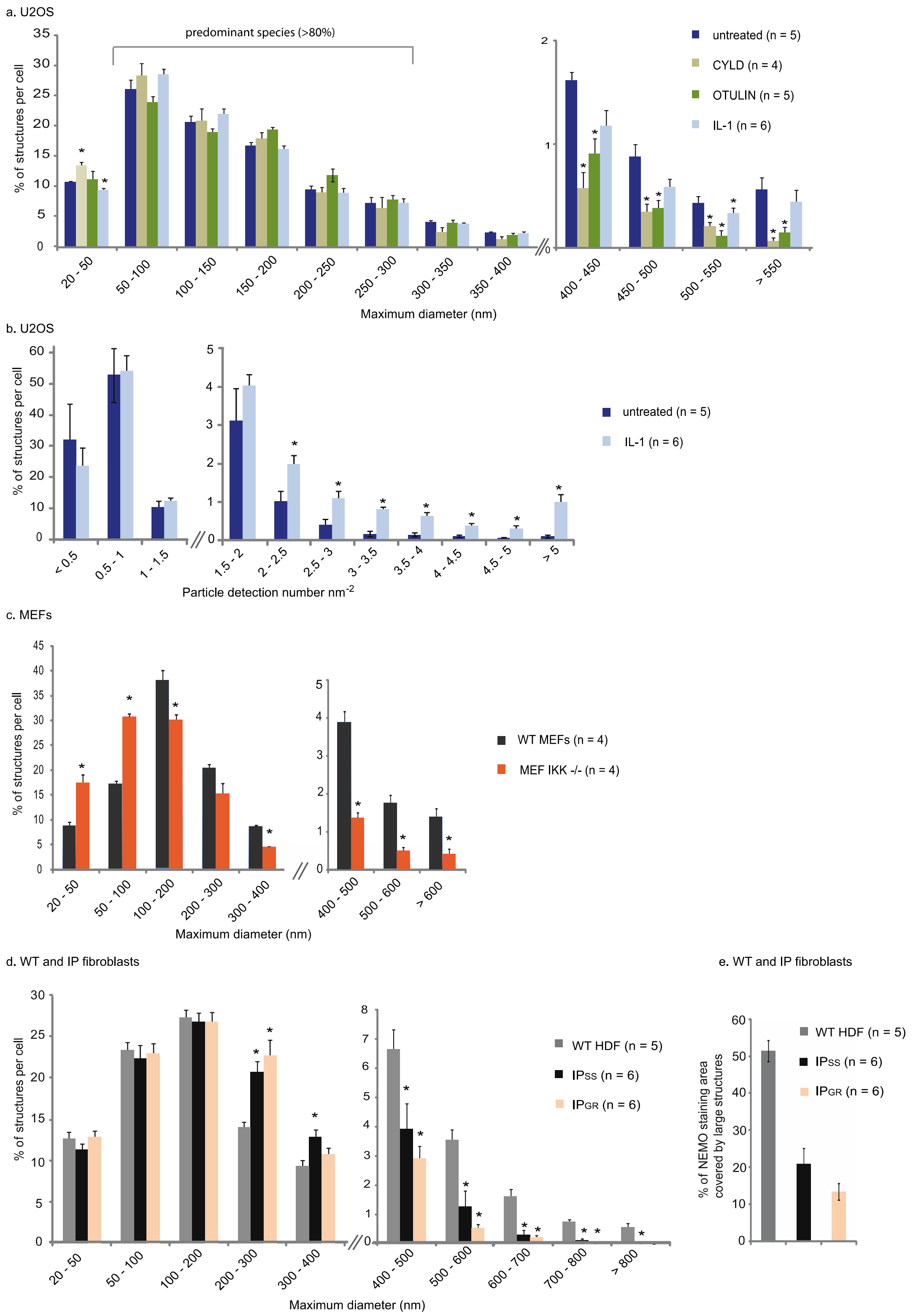

Supplementary Figure 6 Expanded Quantitative analysis.

Quantitative analysis program was performed to analyse the extent and reproducibility of structural differences between conditions and patient cells. a) The maximum diameter of identified structures was compared between untreated, CYLD-, OTULIN over-expressing cells

and cells treated with IL-1. b) The particle density of structures treated with IL-1 was compared with untreated cells. c) The maximum diameter of identified structures was compared between WT MEFs and MEF IKK$^{-/-}$ cells. d) The maximum diameter of identified structures was compared between. e. Assessment of the total NEMO staining area was compared across wild-type, IP_{SS} and IP_{GR} cells. The percentage of the area covered by large NEMO lattice structures over 400 nm in diameter is shown relative to total NEMO staining area. n = number of cell acquisitions. Maximum diameter was binned as indicated on the x axis. Particle density was calculated as the number of particles per nm^2 area occupied by each structure. Quantitative analysis measured number of NEMO structures over 400 nm in diameter per µm^2 of cell area. n is the number of whole cells analysed per condition. * represents a p value of <0.05 after a two-tailed t-test.
